# Trusted Sources of COVID-19 Vaccine Information by County Characteristics in North Carolina

**DOI:** 10.3390/vaccines14010096

**Published:** 2026-01-20

**Authors:** Bryson T. Staley, Michael E. DeWitt, Jennifer J. Wenner, John W. Sanders, Thomas F. Wierzba, Katherine Poehling

**Affiliations:** 1 Biomedical Graduate Programs, Biomedical Sciences, Wake Forest University School of Medicine, Medical Center Boulevard, Winston-Salem, NC 27157, USA; 2 Section on Infectious Diseases, Wake Forest University School of Medicine, Medical Center Boulevard, Winston-Salem, NC 27157, USA; 3 Department of Biology, Wake Forest University, 226 Winston Hall, Box 7325 Reynolda Station, Winston-Salem, NC 27109, USA; 4 Center for Microbial Ecology and Emerging Diseases, Wake Forest University School of Medicine, Medical Center Boulevard, Winston-Salem, NC 27157, USA; 5 Center for Vaccines at the Extremes of Aging, Wake Forest University School of Medicine, Medical Center Boulevard, Winston-Salem, NC 27517, USA; 6 Departments of Pediatrics and Epidemiology and Prevention, Wake Forest University School of Medicine, Medical Center Boulevard, Winston-Salem, NC 27157, USA

**Keywords:** COVID-19 vaccines, urban population, rural population, trust, information sources, vaccination hesitancy

## Abstract

Background/Objectives: The COVID-19 pandemic disproportionately impacted rural areas across the United States, including rural North Carolina (NC). Consistent with national patterns, COVID-19 vaccination coverage as of December 2022 was higher for non-rural (72%) than rural (58%) NC counties. The role of trusted sources of vaccine information used by rural and non-rural residents is unknown. Methods: Using data from two surveys distributed by the COVID-19 Community Research Partnership from 8 June 2021 through 21 December 2021, we compared self-reported sources of trusted COVID-19 vaccine information by non-rural and rural counties and by county-level predominant political vote in the 2020 Presidential election. Results: While NC respondents were highly vaccinated (94%), fewer residents from rural counties self-reported COVID-19 vaccination than those from non-rural counties (91% versus 95%). The most common reported source of trusted vaccine information was federal health agencies. The proportion citing a federal health agency was higher for respondents from non-rural (80%) than rural (72%) counties and was higher for vaccinated (75%) than unvaccinated (42%) rural respondents. The next two most trusted sources of vaccine information were state/local health officials (48%) and health care providers (42%). Among trusted resources reported by 10–15% of respondents, those from rural counties were less likely to use hospital websites, employers, or news sources than those from non-rural counties. More respondents from counties with >60% vote for the 2020 Democratic Presidential candidate cited federal health agencies, state and local officials, and new sources than respondents from counties with >60% vote for the 2020 Republican Presidential candidate. Conclusions: By identifying the trusted sources of vaccine information for residents in non-rural and rural NC counties, future vaccine implementation efforts can tailor communication efforts to increase vaccine uptake and potentially reduce the rates of hospitalizations and death from vaccine-preventable diseases such as COVID-19 or other future pandemics.

## 1. Introduction

From 14 March 2020 through 6 May 2023, North Carolina (NC) recorded 3,501,404 cases and 29,059 deaths attributed to SARS-CoV-2 [1]. Rural NC residents were disproportionately affected as compared to non-rural counterparts [2]; for example, rural deaths were 0.4% and non-rural deaths were 0.2% [3]. Populations from rural counties systematically differ from non-rural counties by being older, having higher rates of co-morbidities, and having more limited access to health care [2]. These differences contribute to an increased vulnerability to illnesses, including COVID-19. From December 2020 through 26 April 2023, 63% of North Carolinians had completed the initial series of COVID-19 vaccination [4] with variations across the 100 NC counties [5]. Notably, vaccine acceptance in NC and the United States varied by race and ethnicity and across rural and non-rural regions [6].

Vaccine hesitancy is multifactorial, with many studies aimed at identifying factors involved in a personal decision to vaccinate or not. A multinational study conducted by the World Health Organization (WHO) examined potential factors associated with vaccine hesitancy by interviewing officials responsible for vaccine dissemination. Results indicated that vaccine hesitancy varies in importance across countries, but vaccine hesitancy can be linked to external factors such as religion, healthcare, and politics, as well as personal beliefs surrounding the safety and efficacy of a vaccine [7]. Specific to the COVID-19 vaccines, previous research by the COVID-19 Community Research Partnership (CCRP) identified self-reported factors behind a personal decision to vaccinate or not. Vaccinated respondents often cited reasons surrounding protecting themselves and those around them from the virus, while unvaccinated respondents often cited concerns about safety and the quickness of vaccine development [8]. Previous studies reported lower vaccination rates among minority groups [6,9]. However, vaccine uptake increased within these populations, who are at an increased risk for poor outcomes, by using tailored surveys and direct contact vaccine information [10].

We built on surveys from the CCPR that demonstrated a positive shift in attitude toward the COVID-19 vaccines across NC’s population. Vaccine uptake increased across all demographic groups regardless of the level of vaccine hesitancy reported at the start of the vaccine rollout [6]. This study is distinct and tests the hypothesis that the sources of trusted information would vary among people residing in rural and non-rural counties and by political leaning (as determined by county vote for the 2020 presidential election). The objective was to test the hypothesis that self-reported sources of trusted vaccine information would differ for respondents from rural and non-rural counties and by respondents who lived in counties that predominantly voted for the Republican candidate or Democratic candidate in the 2020 presidential election.

## 2. Materials and Methods

The COVID-19 Community Research Partnership (CCRP) was a prospective, multi-site, longitudinal study. The design and methods have been described in detail [11]. In summary, the CCRP includes two cohorts, one with six health systems in the mid-Atlantic region and southern USA, and the other with six health systems in North Carolina. Sites invite persons within their health care system, as well as community members, to participate in daily electronic symptoms surveillance, serological testing, risk behavior assessment, and electronic health record capture. Enrollment began in April 2020. Two supplemental vaccine hesitancy surveys in five of 10 CCRP sites, all located in North Carolina (1. Campbell University, 2. New Hanover Regional Medical Center, 3. Wake Forest Baptist Health, 4. WakeMed Health and Hospitals, 5. Vidant Health), were conducted via the CCRP between June and December 2021: one was directed toward participants who were vaccinated and another to those who were unvaccinated [6]. In the CCRP study, each person could respond to both surveys over the 7 months if they were vaccinated between the time the two surveys were distributed. If a respondent answered both surveys, only the first response was included in this analysis. This secondary analysis of the CCRP data set was conducted in accordance with the Declaration of Helsinki and approved by the Institutional Review Board of Wake Forest University School of Medicine (protocol number 109,129 and approved on 20 February 2024).

The survey questions were identical for the vaccinated and unvaccinated surveys. The survey included questions about age, sex, race/ethnicity, level of education, county residence, self-reported vaccination status, level of concern about getting COVID-19 before receiving the COVID-19 vaccine if vaccinated, and up to three trusted sources of information about COVID-19 vaccines. Options for trusted sources of information included community organizations, family and friends, federal health agencies, health insurers, health care providers, health system websites, employers, news sources, online medical websites, professional organizations, religious leaders, social media, state or local health officials, or other.

**Figure 1:**
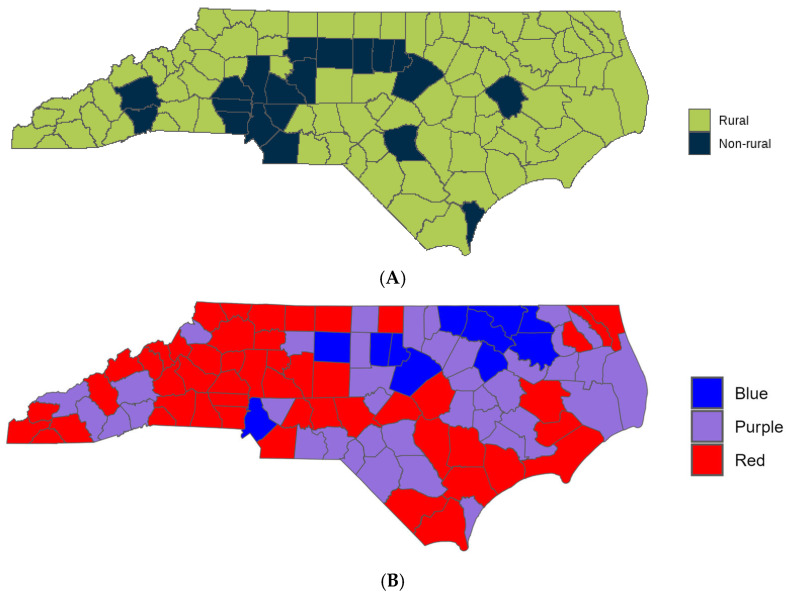
North Carolina County Map by (**A**) rural/non-rural and by (**B**) 2020 Presidential Election Result.

The NC county of residence was self-reported for all participants. County data was evaluated in two ways. (1) Each NC county was classified into rural or suburban/urban (non-rural); most counties in North Carolina are classified as rural with 19 of the 100 counties being classified as non-rural (Figure 1A). (2) The predominant 2020 presidential election vote for each county as a proxy for political leaning of the county (Figure 1B).

County data were classified by the CCRP using methodology from the 2013 NCHS Urban-Rural Classification Scheme for Counties [12] and supplemented by the NC Rural Center based on the 2014 US Census population estimates [13]. This secondary analysis of the CCRP data set utilized the same county classifications used in the initial analysis. Political leaning was determined by the 2020 Presidential election: Counties where >60% voted Democrat are classified as “Blue”, counties where >60% voted Republican are classified as “Red”, and counties where no more than 60% of the votes went toward a single party are classified as “Purple”.

The demographic characteristics of respondents and political leanings of the counties are reported overall and by rural and non-rural NC county of residence. These characteristics are described by counts and proportions for categorical variables and medians and interquartile ranges (IQR) for continuous variables, along with standardized mean differences (SMD) and their corresponding 95% confidence intervals. SMDs were used to assess the magnitude of differences between groups, with values <0.10 considered negligible, 0.10–0.20 small, 0.20–0.50 moderate, and >0.50 large [14].

The primary outcome was self-reported trusted sources of information and was evaluated by each predictor variable (rural versus non-rural county and political leaning of county in 2020 Presidential election). Bivariate analyses were performed by Pearson’s Chi-squared or Fisher’s exact test, and the standardized mean difference was computed. Both the *p*-value of the bivariate analysis and the q-value, which is the *p*-value with false discovery rate correction for multiple testing, are reported [15]. To further elucidate trusted sources of vaccine information among rural respondents, a secondary analysis was performed post hoc.

A secondary outcome was to characterize factors impacting vaccine uptake. A multivariate logistic regression model was performed with reported vaccination status as the dependent variable and with demographic characteristics (age, sex, race/ethnicity), county-level classifications (rural vs. non-rural; 2020 election results categorized as Blue, Purple, or Red), and respondents’ top trusted sources of vaccine information as independent variables. Only information sources selected by at least 200 respondents were retained in the model. The study did not have sufficient power to include interactions in this model. Variance Inflation Factors (VIF) and adjusted VIFs were examined to assess multicollinearity. All predictors had VIFs well below the threshold of 5, indicating low multicollinearity. Standard VIFs ranged from 1.00 to 1.57, and adjusted VIFs ranged from 1.00 to 1.24. The highest VIF was observed for political affiliation (VIF = 1.57, adj. VIF = 1.12) and county classification (VIF = 1.54, adj. VIF = 1.24). Tolerance values ranged from 0.64 to 1.00, further supporting the absence of problematic collinearity.

## 3. Results

A total of 33,815 surveys were obtained from 17,869 respondents between June and December 2021. The June 2021 survey for unvaccinated persons had 1615 responses from 1090 participants. The December 2021 survey for vaccinated persons had 32,200 surveys from 17,101 participants (Table 1). A total of 322 respondents self-reported being both unvaccinated and vaccinated, and the first (unvaccinated) response was included in this analysis. Independent of county of residence, respondents primarily identified as female and white with an average age of 53 (Table 1). Majority of respondents reported having earned a graduate or bachelor’s degree and being “moderately” or “very concerned” about contracting COVID-19 before receiving the vaccine.

Each respondent could select up to 3 of the 14 choices of trusted sources of information about COVID-19; an average of 2.4 trusted sources of information were selected per respondent. The proportion reporting 1, 2, and 3 trusted sources of information was 20.5%, 21.1%, and 58.4%, respectively. The top 3 most trusted sources of information for both rural and non-rural counties (Table 2) were federal agencies (78%), state and local health officials (48%), and health care providers (42%). Trusted sources reported by 10–15% of respondents were employers (14%), news media (12%), medical websites (13%), and hospital websites (10%). All other trusted resources were reported (<10%), with the most frequent selection being family and friends (8%).

Significantly more non-rural than rural respondents reported the following trusted sources of information: federal health agencies, hospital system websites, my employer, and news sources (Table 2). While federal health agencies were the most reported trusted sources of information for all respondents, fewer rural (72%) than non-rural (80%) respondents reported this source. Among the four sources reported by 10–15% of respondents, fewer rural than non-rural respondents selected employers, news media, or hospital websites as a trusted source of information. Among infrequently reported sources (<1%), more rural than non-rural respondents selected religious leaders.

Rural and non-rural counties had a large effect size by the proportion who voted Republican or Democratic in the 2020 Presidential election (Table 3). Most respondents (66%) from rural counties resided in Red counties (voted Republican), whereas most (56%) non-rural respondents resided in Blue counties (voted Democrat).

To further understand the insights of 3141 rural respondents, we evaluated up to three reported trusted sources of information by self-reported vaccination status on the first survey response. The 9.5% who reported being unvaccinated identified an average of 1.7 trusted sources of information about the COVID-19 vaccine; the 90.5% who self-reported being vaccinated identified an average of 2.3 trusted sources of information (Table 2). The top 3 reported trusted sources among rural respondents (Table 4) systematically differed by self-reported vaccination status. The three most common selections for vaccinated respondents were (1) federal agencies, (2) healthcare providers, and (3) state and local health officials. The three most common selections for unvaccinated respondents were (1) federal agencies, (2) healthcare providers, and (3) medical websites. Notably, fewer unvaccinated than vaccinated respondents selected federal health agencies (42% vs. 75%), state and local health officials (16.5% vs. 50%), and health care providers (34% vs. 43%) as a trusted source of information. The unvaccinated respondents reported trusting sources such as online publishers of medical information (20% vs. 14%), professional organizations (8% vs. 5%), social media (3% vs. 1%), and other sources (17.5% vs. 3%) more than vaccinated respondents. The unvaccinated respondents were less likely to report news (4% vs. 12%) and hospital system websites (2% vs. 9%) as trusted sources than vaccinated respondents. There were no significant differences in the frequency of reporting family and friends, employers, or religious leaders.

Among all respondents, 37% resided in Blue (Democratic) counties, 24% in Red (Republican) Counties, and 39% in Purple counties. The most frequently selected trusted sources (Table 5), regardless of political classification, were federal health agencies, state and local health officials, and health care providers. More respondents residing in Blue counties selected federal agencies, state and local health officials, and news sources than respondents residing in Red counties (q < 0.001 for each).

In the multivariable logistic regression analysis for predicting vaccine uptake (Table 6), older age was significantly associated with higher odds of vaccination (OR = 1.04 per year increase in age, 95% CI: 1.03–1.04, *p* < 0.001). Males had 50% greater odds of being vaccinated compared to females (OR = 1.50, 95% CI: 1.27–1.77, *p* < 0.001). Race/ethnicity was also significant: White respondents (OR = 1.74, 95% CI: 1.23–2.40, *p* = 0.001) had higher odds compared to Black respondents. Living in a Red county, but not a Purple county, was associated with substantially lower odds of vaccination (OR = 0.53, 95% CI: 0.43–0.65, *p* < 0.001). Non-rural residence had a non-significant trend compared to rural (OR = 1.19, 95% CI: 0.99–1.44, *p* = 0.065). Trusted sources showed strong associations: respondents who trusted federal health agencies (OR = 4.68), state/local health officials (OR = 3.37), or their employer (OR = 4.09) had markedly higher odds of vaccination (all *p* < 0.001). Trust in healthcare providers (OR = 1.84), news sources (OR = 2.12), hospital system websites (OR = 2.42), and professional organizations (OR = 1.32) also significantly increased odds (*p* < 0.05). Trust in online publishers and family/friends was not significantly associated.

## 4. Discussion

Trusted sources of information across respondents from rural and non-rural counties and by self-reported vaccination status had both commonalities and differences. Overall, the most trusted source of information was federal health agencies, state and local health officials, and health care providers. While federal health agencies were most frequently cited, the proportion reporting this source was higher among respondents who resided in non-rural than rural counties (72% vs. 80%) and among rural respondents who reported being vaccinated than unvaccinated (75% vs. 42%). There were also differences in the trusted sources of information selected by 10–15% of respondents; hospital system websites, employer, and news sources were more frequently reported by non-rural than rural respondents. 2020 presidential election results varied by rural and non-rural counties and respondents who resided in counties that voted Republican in the 2020 Presidential election were less likely to cite federal health agencies, news sources, or state or local health officials than respondents who resided in counties that voted Democratic in the 2020 Presidential election.

Although both political leaning and rurality were associated with vaccination status, the magnitude of effect differed substantially. Residence in Red counties was linked to markedly lower odds of vaccination compared with Blue counties (OR = 0.53, 95% CI 0.43–0.65), while the rural versus non-rural distinction showed only a borderline association (OR = 1.19, 95% CI 0.99–1.44). Beyond these contextual factors, trust in information sources emerged as an even stronger predictor of vaccination: respondents who trusted federal health agencies (OR = 4.68, 95% CI 4.05–5.40), their employer (OR = 4.09, 95% CI 3.14–5.43), or state/local health officials (OR = 3.37, 95% CI 2.82–4.05) had dramatically higher odds of being vaccinated, underscoring the critical role of institutional trust in shaping uptake.

The findings from this study are consistent with previous studies where rural counties had a higher percentage of unvaccinated residents than non-rural counties [8,16]. This study builds on previous research by elucidating differences in trusted sources of information among rural and non-rural counties and the political leaning of a county. Earlier research highlighting the success of the Meningitis Vaccine Project (MVP) and its utilization of a communication strategy including healthcare professionals, local leaders, and members from the target community further emphasizes the importance of identifying key partners in differing NC regions that maximize future vaccine implementation efforts [17].

Respondents from rural and non-rural counties reported similar sources of trusted information regarding COVID-19 with federal agencies (e.g., Centers for Disease Control and Prevention (CDC), NIH, Food and Drug Administration (FDA)), hospital system websites, employers, and news sources being selected most frequently. Federal agencies were the most frequent self-reported source of vaccine information for both rural and non-rural counties. While we measured self-reported trusted sources of information and not trust itself, this finding is consistent with research that reported increased trust in the government increased vaccine acceptance [18]. While rarely reported, religious leaders were more frequently selected as a self-reported trusted source of vaccine information by respondents from rural than non-rural counties. Influences from outside sources, such as social media, reporting contradictory information as well as changing COVID-19 guidelines based upon new data may have caused respondents from rural counties to seek information from sources closer to them [19]. A previous study found that among Evangelicals, clergy who supported the COVID-19 vaccine positively correlated with vaccine uptake amongst their congregation; however, congregants who went to clergy for advice concerning vaccination were less likely to receive the vaccine [20]. This finding may indicate that for some communities partnering with religious leaders could potentially improve future vaccine implementation efforts.

Our results are compatible with previous studies that found a positive relationship between vaccine hesitancy and social media use for vaccine information with increased use of social media use being associated with increased vaccine hesitancy [18,21].

Our results are also compatible with a study showing that Republican confidence in the government declined between 2020 and 2021 and that attitudes surrounding government trust vary based on the political party in power [22]. We found that respondents who resided in counties that voted >60% Democrat for the 2020 Presidential election were more likely to trust information from federal agencies, news sources, and state or local health officials than respondents who resided in counties that voted >60% Republican for the 2020 Presidential election. While respondents from rural counties predominantly voted Republican in the 2020 presidential election and were less likely to be vaccinated, rural counties vary and have many different influences affecting a person’s decision to vaccinate or not. Taking the political leaning of each county into accoun may influence the trusted sources of information and help design future public health outreach efforts.

This study has several limitations. Given that the respondents of this study self-reported being primarily 42–64 years of age, female, white, and possessing a bachelor’s degree or higher, the results of this study may not be generalizable to the greater NC population or the US population. Since respondents were already connected to a healthcare system, they may have been more comfortable responding to a survey from a healthcare system. The information collected was self-reported and so social desirability biases are possible. This study classified each NC county as rural or non-rural as that was the most granular data available, however many NC counties contain both rural and non-rural regions within the county. The political leaning of each county was determined by the predominant vote for the 2020 Presidential election just before the onset of the COVID-19 pandemic. The results may vary if other political measures were assessed. We compared proportions, which implicitly acknowledges the variability within each county, and acknowledge that not every person who resides in a County voted or shares the same opinions as the majority. Respondents from both rural and non-rural counties were combined to assess trusted sources of information with political leaning because of the significant overlap in rural and non-rural counties and preponderant county vote for 2020 Presidential election, we cannot differentiate between the influence of county political leaning and county rurality.

## 5. Conclusions

In conclusion, this study examines self-reported trusted sources of COVID-19 vaccine information from residents of both rural and non-rural NC counties and compares it to respondents’ vaccination status. By 2023, most adults in NC were fully vaccinated, emphasizing the efficiency in utilizing institutions such as hospital systems, federal agencies, and news outlets for vaccine information dissemination. However, rural populations reported lower vaccination rates despite experiencing worse outcomes from the pandemic. Similar to previous studies, results from this study identify federal health agencies as the most common source of trusted vaccine information, though less frequently selected by rural respondents. Rural counties tended to lean Republican in the 2020 Presidential election, indicating a potential political element in vaccine hesitancy, yet the extent of its effect is unknown. By identifying trusted sources of vaccine information in rural communities, future vaccine implementation efforts can be tailored to rural communities in an effort increase vaccine uptake.

## Figures and Tables

**Table 1:** Characteristics, COVID-19 concern, & Vaccine Status of 17,869 Respondents in the 6-month surveys.

		County Classification			
Characteristic	Overall *N* = 17,869 ^1^	Rural *N* = 3141 ^1^	Non-Rural *N* = 14,728 ^1^	Difference ^2^	95% CI ^2^
Age, years	53 (42, 64)	56 (44, 65)	53 (41, 63)	0.16	0.12, 0.20
Sex				0.10	0.06, 0.14
Female	13,136 (73.5%)	2420 (77.1%)	10,716 (72.8%)		
Male	4731 (26.5%)	720 (22.9%)	4011 (27.2%)		
Unknown	2	1	1		
Race/Ethnicity				0.08	0.04, 0.12
Black	561 (3.1%)	78 (2.5%)	483 (3.3%)		
Hispanic or Latino	273 (1.5%)	29 (0.9%)	244 (1.7%)		
White	16,539 (92.6%)	2953 (94.0%)	13,586 (92.2%)		
Other or Unspecified	496 (2.8%)	81 (2.6%)	415 (2.8%)		
Highest Education Level				0.38	0.34, 0.42
Less than high school	24 (0.1%)	6 (0.2%)	18 (0.1%)		
High school or equivalent	591 (3.5%)	178 (6.0%)	413 (2.9%)		
Some college but no degree	1731 (10.2%)	420 (14.2%)	1311 (9.3%)		
Associate	1697 (10.0%)	481 (16.3%)	1216 (8.6%)		
Bachelor	5684 (33.4%)	931 (31.5%)	4753 (33.8%)		
Graduate	7301 (42.9%)	940 (31.8%)	6361 (45.2%)		
Unknown	841	185	656		
Before receiving a COVID-19 vaccine, how concerned were you about getting COVID-19?				0.03	−0.01, 0.07
Not at all concerned	2291 (12.8%)	380 (12.1%)	1911 (13.0%)		
A little concerned	3390 (19.0%)	606 (19.3%)	2784 (18.9%)		
Moderately concerned	4803 (26.9%)	829 (26.4%)	3974 (27.0%)		
Very concerned	7385 (41.3%)	1326 (42.2%)	6059 (41.1%)		
Vaccination Status				0.17	0.13, 0.21
Unvaccinated	1051 (5.9%)	297 (9.5%)	754 (5.1%)		
Vaccinated	16,818 (94.1%)	2844 (90.5%)	13,974 (94.9%)		

^1^ Median (IQR) or Frequency (%). ^2^ Standardized Mean Difference, no large effects (|SMD| ≥ 0.5). Abbreviations: CI = Confidence Interval; IQR = Interquartile Range.

**Table 2:** Trusted Sources of Vaccine Information by Rural or Non-Rural County of Residence of 17,869 Respondents in the 6-month Surveys.

	County Classification					
Characteristic	Rural *N* = 3141 ^1^	Non-Rural *N* = 14,728 ^1^	Difference ^2^	95% CI ^2^	*p*-Value ^3^	q-Value ^4^
Community organizations	13 (0.4%)	35 (0.2%)	0.03	−0.01, 0.07	0.083	0.15
Family and friends	258 (8.2%)	1190 (8.1%)	0.00	−0.03, 0.04	0.8	0.9
Federal health agencies (e.g., Centers for Disease Control and Prevention (CDC), Food and Drug Administration (FDA), National Institutes of Health (NIH))	2271 (72.3%)	11,747 (79.8%)	−0.18	−0.21, −0.14	<0.001	**<0.001**
Health insurers	40 (1.3%)	145 (1.0%)	0.03	−0.01, 0.07	0.15	0.2
Healthcare providers	1319 (42.0%)	6098 (41.4%)	0.01	−0.03, 0.05	0.5	0.6
Hospital system websites (e.g., Kaiser Permanente)	265 (8.4%)	1482 (10.1%)	−0.06	−0.09, −0.02	0.005	**0.025**
My employer	374 (11.9%)	2100 (14.3%)	−0.07	−0.11, −0.03	<0.001	**0.004**
News sources	341 (10.9%)	1851 (12.6%)	−0.05	−0.09, −0.01	0.008	**0.028**
Online publishers of medical information (e.g., WebMD, Mayo Clinic)	446 (14.2%)	1896 (12.9%)	0.04	0.00, 0.08	0.046	0.091
Other	140 (4.5%)	664 (4.5%)	0.00	−0.04, 0.04	0.9	0.9
Professional organizations	159 (5.1%)	887 (6.0%)	−0.04	−0.08, 0.00	0.037	0.087
Religious leaders	30 (1.0%)	84 (0.6%)	0.04	0.01, 0.08	0.014	**0.039**
Social media	35 (1.1%)	137 (0.9%)	0.02	−0.02, 0.06	0.3	0.4
State or local health officials	1464 (46.6%)	7046 (47.8%)	−0.02	−0.06, 0.01	0.2	0.3

^1^ Frequency (%). ^2^ Standardized Mean Difference, no large effects (|SMD| ≥ 0.5). ^3^ Pearson’s Chi-squared test. ^4^ False discovery rate correction for multiple testing, significant values in bold (q < 0.05). Abbreviation: CI = Confidence Interval.

**Table 3:** County Political Leaning by Rural or Non-rural County of Residence of 17,869 Respondents in the 6-month Surveys.

		County Classification			
Characteristic	Overall *N* = 17,869 ^1^	Rural *N* = 3141 ^1^	Non-Rural *N* = 14,728 ^1^	Difference ^2^	95% CI ^2^
Red (Voted Republican, %)	42 (36, 58)	66 (60, 75)	42 (36, 42)	**2.2**	2.2, 2.3
Blue (Voted Democrat, %)	56 (41, 62)	33 (24, 38)	56 (56, 62)	**−2.2**	−2.2, −2.2

^1^ Median (IQR). ^2^ Standardized Mean Difference, large effects in bold (|SMD| ≥ 0.5). Abbreviations: CI = Confidence Interval; IQR = Interquartile Range. All *p*-values and q-values < 0.001.

**Table 4:** Trusted Sources of Vaccine Information by Vaccination Status of 3141 Rural Respondents in the 6-month surveys.

	Vaccination Status					
Characteristic	Unvaccinated *N* = 297 ^1^	Vaccinated *N* = 2844 ^1^	Difference ^2^	95% CI ^2^	*p*-Value ^3^	q-Value ^4^
Community organizations	2 (0.7%)	11 (0.4%)	0.04	−0.08, 0.16	0.4	0.4
Family and friends	33 (11.1%)	225 (7.9%)	0.11	−0.01, 0.23	0.056	0.074
Federal health agencies (e.g., Centers for Disease Control and Prevention (CDC), Food and Drug Administration (FDA), National Institutes of Health (NIH))	126 (42.4%)	2145 (75.4%)	**−0.71**	−0.83, −0.59	<0.001	**<0.001**
Health insurers	5 (1.7%)	35 (1.2%)	0.04	−0.08, 0.16	0.4	0.4
Healthcare providers	101 (34.0%)	1218 (42.8%)	−0.18	−0.30, −0.06	0.003	**0.007**
Hospital system websites (e.g., Kaiser Permanente)	8 (2.7%)	257 (9.0%)	−0.27	−0.39, −0.15	<0.001	**<0.001**
My employer	27 (9.1%)	347 (12.2%)	−0.10	−0.22, 0.02	0.12	0.13
News sources	12 (4.0%)	329 (11.6%)	−0.28	−0.40, −0.16	<0.001	**<0.001**
Online publishers of medical information (e.g., WebMD, Mayo Clinic)	59 (19.9%)	387 (13.6%)	0.17	0.05, 0.29	0.003	**0.007**
Other	52 (17.5%)	88 (3.1%)	0.49	0.37, 0.61	<0.001	**<0.001**
Professional organizations	24 (8.1%)	135 (4.7%)	0.14	0.02, 0.26	0.013	**0.020**
Religious leaders	6 (2.0%)	24 (0.8%)	0.10	−0.02, 0.22	0.058	0.074
Social media	9 (3.0%)	26 (0.9%)	0.15	0.03, 0.27	0.004	**0.007**
State or local health officials	49 (16.5%)	1415 (49.8%)	**−0.76**	−0.88, −0.63	<0.001	**<0.001**

^1^ Frequency (%). ^2^ Standardized Mean Difference, large effects in bold (|SMD| ≥ 0.5). ^3^ Fisher’s exact test; Pearson’s Chi-squared test. ^4^ False discovery rate correction for multiple testing, significant values in bold (q < 0.05). Abbreviation: CI = Confidence Interval.

**Table 5:** Trusted Sources of Vaccine Information by County Political Leaning of 17,869 Respondents in the 6-month Surveys.

	County of Residence Political Leaning						
Characteristic	Blue *N* = 6678 ^1^	Purple *N* = 6928 ^1^	Red *N* = 4263 ^1^	Difference Between Blue vs. Red ^2^	95% CI ^2^	*p*-Value ^3^	q-Value ^4^
Community organizations	13 (0.2%)	21 (0.3%)	14 (0.3%)	−0.03	−0.06, 0.01	0.2	0.3
Family and friends	514 (7.7%)	599 (8.6%)	335 (7.9%)	−0.01	−0.04, 0.03	0.8	0.9
Federal health agencies (e.g., Centers for Disease Control and Prevention (CDC), Food and Drug Administration (FDA), National Institutes of Health (NIH))	5480 (82.1%)	5464 (78.9%)	3074 (72.1%)	0.24	0.20, 0.28	<0.001	**<0.001**
Health insurers	68 (1.0%)	59 (0.9%)	58 (1.4%)	−0.03	−0.07, 0.01	0.10	0.2
Healthcare providers	2782 (41.7%)	2843 (41.0%)	1792 (42.0%)	−0.01	−0.05, 0.03	0.7	0.9
Hospital system websites (e.g., Kaiser Permanente)	650 (9.7%)	681 (9.8%)	416 (9.8%)	0.00	−0.04, 0.04	>0.9	>0.9
My employer	927 (13.9%)	917 (13.2%)	630 (14.8%)	−0.03	−0.06, 0.01	0.2	0.3
News sources	903 (13.5%)	902 (13.0%)	387 (9.1%)	0.14	0.10, 0.18	<0.001	**<0.001**
Online publishers of medical information (e.g., WebMD, Mayo Clinic)	885 (13.3%)	889 (12.8%)	568 (13.3%)	0.00	−0.04, 0.04	>0.9	>0.9
Other	275 (4.1%)	333 (4.8%)	196 (4.6%)	−0.02	−0.06, 0.01	0.2	0.4
Professional organizations	444 (6.6%)	358 (5.2%)	244 (5.7%)	0.04	0.00, 0.08	0.052	0.15
Religious leaders	38 (0.6%)	36 (0.5%)	40 (0.9%)	−0.04	−0.08, 0.00	0.025	0.088
Social media	64 (1.0%)	73 (1.1%)	35 (0.8%)	0.01	−0.02, 0.05	0.5	0.6
State or local health officials	3239 (48.5%)	3414 (49.3%)	1857 (43.6%)	0.10	0.06, 0.14	<0.001	**<0.001**

^1^ Frequency (%). ^2^ Standardized Mean Difference, no large effects (|SMD| ≥ 0.5). ^3^ Pearson’s Chi-squared test. ^4^ False discovery rate correction for multiple testing, significant values in bold (q < 0.05). Abbreviation: CI = Confidence Interval.

**Table 6:** Multivariate Logistic Regression of Vaccine Update.

		County Classification	
Characteristic	OR	95% CI	*p*-Value ^1^
Age, years	1.04	1.03, 1.04	**<0.001**
Sex			
Female	-	-	
Male	1.50	1.27, 1.77	**<0.001**
Race/Ethnicity			
Black	-	-	
Hispanic or Latino	1.87	1.00, 3.65	0.057
White	1.74	1.23, 2.40	**0.001**
Other or Unspecified	2.17	1.27, 3.77	**0.005**
County Rural Classification			
Rural	-	-	
Non-rural	1.19	0.99, 1.44	0.065
County 2020 Election Classification			
Blue	-	-	
Purple	0.89	0.75, 1.05	0.2
Red	0.53	0.43, 0.65	**<0.001**
Trusted Sources of Vaccine Information			
Federal health agencies (e.g., Centers for Disease Control and Prevention (CDC), Food and Drug Administration (FDA), National Institutes of Health (NIH))	4.68	4.05, 5.40	**<0.001**
State or local health officials	3.37	2.82, 4.05	**<0.001**
Healthcare providers	1.84	1.60, 2.11	**<0.001**
My employer	4.09	3.14, 5.43	**<0.001**
Online publishers of medical information (e.g., WebMD, Mayo Clinic)	1.01	0.85, 1.20	>0.9
News sources	2.12	1.64, 2.78	**<0.001**
Hospital system websites (e.g., Kaiser Permanente)	2.42	1.82, 3.29	**<0.001**
Family and friends	1.17	0.95, 1.46	0.15
Professional Organizations	1.32	1.03, 1.71	**0.031**

^1^ significant values in bold (*p* < 0.05). Abbreviations: CI = Confidence Interval; OR = Odds Ratio.

## Data Availability

Results of the COVID-19 CRP are being disseminated on the study website (https://www.covid19communitystudy.org/ accessed 18 January 2026) as well as in publications and presentations in medical journals and at scientific meetings. At end of the study, the databases will be made publicly available in a de-identified manner according to CDC and applicable U.S. Federal policies.

## Abbreviations

The following abbreviations are used in this manuscript:

NC	North Carolina
WHO	World Health Organization
CCRP	COVID-19 Community Research Partnership
IQR	Interquartile Ranges
SMD	Standardized Mean Differences
MVP	Meningitis Vaccine Project
CDC	Center for Disease Control and Prevention
NIH	National Institutes of Health
FDA	Food and Drug Administration

## Appendix A

Thank you for being a part of the COVID-19 Community Research Partnership.

We want to learn about how attitudes toward COVID-19 vaccines change over time. To do this, we would like to ask you to complete a brief survey each month.

Preliminary question: Have you received one or more doses of a COVID-19 vaccine?

○ No; I have not received any doses of a COVID-19 vaccine
○ Yes; I have received one or more doses of a COVID-19 vaccine
○ Not sure; I am currently participating in a COVID-19 vaccine trial

[If No, Unvaccinated Questionnaire]

[If Yes or Not sure, Vaccinated Questionnaire]

○ I will complete the survey now
○ Remind me tomorrow
○ I am not interested in taking part in this survey at this time

(Survey Questions: Adults who have not received at least one dose of vaccination:)

1. How concerned are you about getting COVID-19?
  ○ Not at all concerned
  ○ A little concerned
  ○ Moderately concerned
  ○ Very concerned
2. If a COVID-19 vaccine were available to you, would you get it?
  ○ Yes, I would get it as soon as possible
  ○ Yes, but I plan to wait to get it
  ○ Not sure
  ○ No
3. If you have one or more children under 18 years old, how likely are you to get them a COVID-19 vaccine when they are eligible?
  ○ Not at all likely
  ○ Somewhat likely
  ○ Extremely likely
  ○ They are already vaccinated
  ○ N/A
4. Which of the following COVID-19 vaccines would you be willing to receive? (Select all that apply)
  ○ Pfizer-BioNTech
  ○ Moderna
  ○ Johnson & Johnson/Janssen
  ○ AstraZeneca
  ○ Novavax
  ○ Other
  ○ None of the above
5. With which of the following statements about COVID-19 vaccines do you agree? (Select all that apply)
  ○ I am concerned about possible side effects
  ○ Getting vaccinated would make me feel safe resuming my pre-COVID-19 activities
  ○ I am concerned that the vaccines were developed too quickly
  ○ Getting vaccinated is a good way to protect myself from getting sick with COVID-19
  ○ I have ethical concerns about the vaccines
  ○ I do not trust the science behind the vaccines
  ○ I do not need a COVID-19 vaccine to keep me healthy
  ○ Getting vaccinated is important for the health of my community
  ○ I am concerned about the safety of the vaccines
  ○ None of these apply
  ○ Not sure
6. At this point in time, how easy do you think it will be to get a COVID-19 vaccine?
  ○ Very easy
  ○ Somewhat easy
  ○ Somewhat difficult
  ○ Very difficult
  ○ Not sure
7. If you think getting a COVID-19 vaccine will not be very easy, what might make it difficult for you to get a COVID-19 vaccine? (Select all that apply. If you think getting a COVID-19 vaccine will be very easy, please select N/A)
  ○ I have a medical reason that makes me ineligible to get vaccinated (e.g., I have had a severe allergy to vaccines in the past)
  ○ It is difficult to find or make an appointment
  ○ It is difficult to get to the vaccination site
  ○ The hours of operation are inconvenient
  ○ The waiting time is too long
  ○ It is difficult to arrange for childcare
  ○ It is difficult to get time off work
  ○ Other
  ○ I do not plan to get vaccinated
  ○ N/A
8. What would motivate you the most to get vaccinated? (Select up to three)
  ○ To protect my health
  ○ To protect health of people I care about (e.g., family, friends, co-workers, community)
  ○ To get back to work/school
  ○ To get the economy back on track
  ○ To prevent spread of new variants or strains
  ○ To resume social activities
  ○ Receiving rewards or entry into a lottery
  ○ To resume travel
  ○ My healthcare provider recommending that I get vaccinated
  ○ Local leaders (e.g., local representative, religious leader) encouraging me to get vaccinated
  ○ Other
  ○ Not sure
  ○ Nothing would motivate me to get vaccinated
9. In speaking with your healthcare provider, which of the following most closely matches their recommendation about COVID-19 vaccines?
  ○ Strongly discouraged
  ○ Discouraged
  ○ Encouraged
  ○ Strongly encouraged
  ○ Encouraged, but with modified recommendations
  ○ I have not spoken with a healthcare provider about COVID-19 vaccines
10. What are your most trusted sources of information about COVID-19 vaccines? (Select up to three)
  ○ Federal health agencies (e.g., Centers for Disease Control and Prevention (CDC), Food and Drug Administration (FDA), National Institutes of Health (NIH))
  ○ State or local health officials
  ○ My employer
  ○ Family and friends
  ○ Health insurers
  ○ Hospital system websites (e.g., Kaiser Permanente)
  ○ Healthcare providers
  ○ News sources
  ○ Professional organizations
  ○ Community organizations
  ○ Religious leaders
  ○ Online publishers of medical information (e.g., WebMD, Mayo Clinic)
  ○ Social media
  ○ Other
11. Do you know anyone who has received at least one dose of a COVID-19 vaccine? (Select all that apply)
  ○ Yes, someone in my household
  ○ Yes, most adults in my household
  ○ Yes, at least one eligible child (less than 18 years old) in my household
  ○ Yes, someone outside my household I spend time with each week
  ○ Yes, most adults outside my household I spend time with each week
  ○ Yes, someone I personally know but do not spend time with each week
  ○ Not sure, I don’t know whether people I know have been vaccinated
  ○ No, I don’t personally know anyone who has been vaccinated
